# Metabolic Reprogramming-Driven Lactylation: Emerging Mechanisms Linking DNA Damage Repair and Chemoresistance in Cancer

**DOI:** 10.3390/cells15121073

**Published:** 2026-06-13

**Authors:** Lining Wang, Siyu Zhong, Jianan Zhao, Ligang Liu, Changyong Li

**Affiliations:** 1 School of Public Health, Qilu Medical University, Zibo 255300, China; wanglining@qlmu.edu.cn; 2 Institute of Rehabilitation Medicine, Qilu Medical University, Zibo 255300, China; 17658612814@163.com; 3 Graduate School of Shanghai University of Traditional Chinese Medicine, Shanghai 201203, China; aaronliver@163.com; 4 Department of Pharmacy Practice and Administration, College of Pharmacy, Western University of Health Sciences, Pomona, CA 91766, USA

**Keywords:** lactylation, DNA damage repair, tumour metabolism, chemoresistance, lactate

## Abstract

Lactylation is an emerging lactate-derived post-translational modification that may link tumour metabolic reprogramming, epigenetic regulation and DNA damage repair. Enhanced glycolysis and lactate accumulation are common in many tumours, and lactate has been reported to induce histone and non-histone lactylation in specific experimental contexts. Recent studies suggest that lactylation is associated with several DNA repair pathways, including base excision repair/single-strand break repair, nucleotide excision repair, homologous recombination and non-homologous end joining, and may contribute to therapy resistance in selected cancer models. Specifically, XRCC1 lactylation has been reported to promote nuclear translocation and repair activity in glioblastoma models; H4K12 lactylation has been linked to PARP inhibitor resistance through RAD23A activation in ovarian cancer models; and BLM lactylation has been associated with enhanced homologous recombination repair in bladder cancer models. Lactylation of NBS1, RAD51 and XLF has also been implicated in DNA repair regulation in specific experimental systems, although some mechanistic links are inferred from pathway activation or functional rescue experiments rather than directly demonstrated across multiple tumour types. These findings suggest that lactylation may modulate DNA repair and therapeutic response in a context-dependent manner. Targeting lactate metabolism, transport and lactylation regulators, including LDHA, MCT1/4, ACAT1, AARS1 and GCN5, or using site-specific lactylation-inhibiting peptides may improve chemotherapy and PARP inhibitor efficacy, but clinical translation remains limited by heterogeneity, metabolic plasticity, toxicity and insufficient validation.

## 1. Introduction

The accurate expression of genomic DNA is essential for all biological processes within the body. However, cellular genomic DNA is under constant attack, with an average of 10^4^–10^5^ spontaneous DNA damage events occurring in a single cell every day, posing a serious threat to genomic stability [1]. Various endogenous factors such as DNA replication stress, reactive oxygen species (ROS) (generated by physiological processes such as cellular respiration) and lipid peroxidation and a range of exogenous factors including ultraviolet (UV) radiation, ionising radiation, genotoxic chemicals and viruses can all cause damage to DNA bases or the molecular structure of DNA. Types of DNA damage include base insertions/deletions, mismatches, interstrand cross-links, single-strand breaks (SSBs) and double-strand breaks (DSBs). To address these damages, eukaryotes have evolved a highly conserved DNA damage response (DDR) system. The core function of the DDR system is to recognise and repair damaged DNA whilst regulating biological processes such as the cell cycle, chromatin remodelling, gene transcription and cell death. This maintains genomic stability whilst enhancing the cell’s tolerance to DNA damage [2,3]. DDR defects lead to genomic instability, which is not only a major driver of tumourigenesis but also constitutes a potential vulnerability that can serve as a target for cancer therapy [4,5].

Lactate, a by-product of glycolysis, was once regarded as a waste product of cellular metabolism. However, growing evidence suggests that lactate influences a variety of physiological and pathological processes, exerting a wide range of biological effects [6]. The key roles of lactate in metabolism include serving as an energy source, a precursor for gluconeogenesis, and a signalling molecule involved in signal transduction. Lactate may act as a “shuttle” within and between cells, primarily mediated by monocarboxylate transporters (MCTs) [7,8].

Within the tumour microenvironment (TME), the function of lactate undergoes a fundamental shift. Even under conditions of sufficient oxygen, tumour cells prioritise rapid energy production via glycolysis, generating large amounts of lactate—a phenomenon known as the Warburg effect. The Warburg effect is not only metabolically significant; its impact extends far beyond metabolism itself, profoundly regulating a variety of biological processes, including mitochondrial function, gene transcription, the cell cycle, DNA damage repair, and tumour immune evasion.

Post-translational modifications (PTMs) are one of the key mechanisms regulating cellular biological functions, capable of modulating protein stability, activity, structure, localisation, and interactions between proteins and other biomolecules. PTMs can occur on both histones and non-histones; these modifications confer new properties on proteins to respond to environmental changes or growth requirements [9]. With the advancement of proteomics technologies, hundreds of different types of PTMs have been observed in various proteins across eukaryotes, bacteria and archaea [10,11]. Classic PTMs include phosphorylation, acetylation, methylation and ubiquitination. In 2019, Zhang et al. first identified a novel histone modification—lysine lactylation. This modification regulates gene transcription through epigenetic mechanisms, establishing a direct link between cellular metabolic state and gene expression regulation [12]. Subsequent studies have further confirmed that histone lactylation is involved in regulating various key cellular functions, such as glycolysis and macrophage polarisation, and plays a significant role in the pathophysiological processes of numerous diseases, including cancer, thereby providing a novel perspective for investigating the mechanisms by which metabolic reprogramming drives disease progression.

Many classic chemotherapeutic agents, such as platinum compounds, anthracyclines, alkylating agents and topoisomerase inhibitors, exert their antitumour effects primarily by inducing DNA cross-links, single-strand breaks or double-strand breaks. However, tumour cells can develop resistance to chemotherapy by enhancing DNA repair pathways—such as base excision repair (BER), nucleotide excision repair (NER), homologous recombination (HR) and non-homologous end-joining (NHEJ)—to repair drug-induced DNA damage. Recent studies have shown that lactate accumulation driven by tumour metabolic reprogramming not only promotes acidification of the tumour microenvironment but also directly regulates the function of DDR-related proteins and chromatin structure through lactylation modifications, thereby reshaping the DNA damage repair network and promoting treatment tolerance. This article aims to systematically summarise the metabolic basis of lactylation and its enzymatic regulatory mechanisms, with a focus on elucidating the potential roles of lactylation in BER, NER, HR and NHEJ, as well as its mechanisms and therapeutic prospects in cancer chemotherapy resistance.

Compared with previous reviews that have broadly discussed the roles of lactylation in cancer progression and therapy resistance [13], this review provides several additional perspectives. First, we organise the available evidence according to individual DNA repair pathways, including BER/SSB repair, NER, HR and NHEJ, thereby providing a pathway-by-pathway framework for understanding how lactylation may influence DDR regulation. Second, we summarise reported lactylation-related targets, tumour models, associated repair pathways and therapeutic implications in a dedicated table, which helps clarify the context and strength of current evidence. Third, we incorporate recently published studies, including newly reported histone and non-histone lactylation events related to DNA repair and treatment resistance. Together, these updates provide a more structured and current overview of lactylation-mediated regulation of DNA repair in cancer.

This narrative review was based on a literature search of PubMed, Web of Science and Google Scholar up to March 2026. The search terms included combinations of “lactylation”, “lysine lactylation”, “histone lactylation”, “non-histone lactylation”, “lactate metabolism”, “tumour metabolism”, “DNA damage response”, “DNA damage repair”, “base excision repair”, “single-strand break repair”, “nucleotide excision repair”, “homologous recombination”, “non-homologous end joining”, “PARP inhibitor resistance”, “chemotherapy resistance” and “cancer”. Additional relevant studies were identified from the reference lists of selected articles. We mainly included original research articles and recent reviews related to lactylation, tumour metabolism, DNA damage repair and therapy resistance.

## 2. The Molecular Basis of Lactate-Mediated Regulation: From Tumour Metabolism to Epigenetic Regulation

### 2.1. Lactate Production

Lactate is the end product of glycolysis, a key metabolic pathway by which cells break down glucose to produce lactate and energy under hypoxic conditions [14]. Traditionally, lactate has been regarded as a metabolic waste product that is broken down by the liver and subsequently converted back into glucose via gluconeogenesis [15,16].

In addition to acting as a metabolic by-product, lactate can also regulate gene expression via histone lactylation. Lactate metabolism is closely associated with lipid reprogramming and can support the synthesis of fatty acids, cholesterol and membrane lipids via the pyruvate and acetyl-CoA pathways. Concurrently, enhanced glycolysis promotes activation of the pentose phosphate pathway, supplying ribose-5-phosphate and NADPH for nucleotide synthesis, and influences DNA/RNA modification by altering the levels of metabolites such as SAM, α-KG, acetyl-CoA and NAD^+^. Consequently, lactate metabolism links energy metabolism, lipid modification, nucleotide synthesis and epigenetic regulation, serving as a crucial regulatory hub in disease progression and treatment tolerance.

#### 2.1.1. Lactate Production in Normal Cells

**Figure 1:**
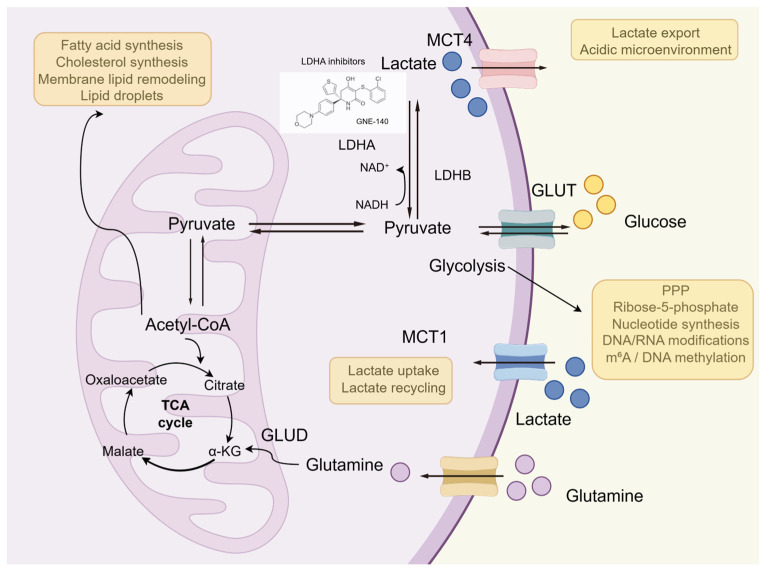
Schematic diagram of lactate formation, metabolism and associated lipid and nucleotide modification mechanisms. In tumour cells, glucose is taken up via GLUT and enters glycolysis, producing pyruvate; some of the pyruvate enters the mitochondria to form acetyl-CoA and participate in the tricarboxylic acid (TCA) cycle, whilst the remainder is converted to lactate by LDHA. Lactate can be exported via MCT4 or re-uptaken via MCT1; simultaneously, glutamine metabolism replenishes α-ketoglutarate via GLUD, supporting the TCA cycle. This diagram summarises the fundamental processes of lactate production, transport and coupling with mitochondrial metabolism in tumour cells. Abbreviations: MCT1, monocarboxylate transporter 1; MCT4, monocarboxylate transporter 4; GLUT, glucose transporter; LDHA, lactate dehydrogenase A; LDHB, lactate dehydrogenase B; GLUD, glutamate dehydrogenase; TCA, tricarboxylic acid; α-KG, α-ketoglutarate; Acetyl-CoA, acetyl coenzyme A.

Under normal conditions, lactate primarily exists in two isomers: L-lactate and D-lactate. L-lactate, the predominant lactate isomer in mammalian cells, is mainly produced from pyruvate through glycolysis. In contrast, D-lactate is primarily generated through the methylglyoxal pathway. In this pathway, methylglyoxal (MGO), a reactive by-product of glycolysis, is converted to S-D-lactoylglutathione by glyoxalase 1 (GLO1) in the presence of glutathione and subsequently hydrolysed by glyoxalase 2 (GLO2) to produce D-lactate [17]. Under hypoxic conditions, pyruvate can be reduced to lactate by LDHA to regenerate NAD^+^ and sustain the continuation of glycolysis. In contrast, tumour cells can convert large amounts of pyruvate into lactate even under conditions of sufficient oxygen; this phenomenon is known as aerobic glycolysis or the Warburg effect [18]. Lactate metabolism and the lactate shuttle are illustrated in Figure 1.

#### 2.1.2. Lactate Production in Tumour Cells

Cancer cells exhibit unique metabolic characteristics distinct from those of normal cells. In the 1920s, Otto Warburg first discovered the phenomenon of aerobic glycolysis in tumour tissue, known as the Warburg effect. This metabolic alteration not only promotes tumour cell progression and metastasis but also exacerbates the accumulation of lactate in solid tumours [19].

Within the tumour microenvironment, even when oxygen supply is relatively adequate, tumour cells still tend to produce large amounts of lactate via glycolysis. This lactate not only provides energy and biosynthetic substrates for tumour cells but also, by acidifying the microenvironment, promotes extracellular matrix degradation, tumour invasion and metastasis, immune suppression and angiogenesis, thereby creating favourable conditions for the progression of malignant tumours [20].

Excess lactate is transported out of the cells, leading to acidification of TME, which in turn degrades the extracellular matrix and promotes tumour invasion and metastasis [21]. Furthermore, the acidic environment suppresses immune cell function, helping cancer cells evade the immune response [22]. During glycolysis, glucose is converted into pyruvate; approximately 85% of this pyruvate is converted into lactate under the catalysis of LDH, even under conditions of sufficient oxygen supply. Only a small proportion (approximately 5%) of pyruvate enters the mitochondria to participate in the tricarboxylic acid (TCA) cycle for ATP production [21]. Furthermore, cancer cells can further increase glycolytic flux by upregulating glucose transporter expression and activating signalling pathways to meet the energy demands of rapid proliferation [23].

Multiple oncogenes and tumour suppressor genes are involved in regulating the glycolytic reprogramming of tumour cells. For example, oncogenes such as MYC and RAS can enhance glycolysis, whilst tumour suppressor genes that typically inhibit glycolysis (such as p53) are frequently mutated or inactivated in cancer. Under hypoxic tumour conditions, hypoxia-inducible factor (HIF)-1 becomes stabilised, thereby activating genes associated with glycolysis, including those encoding glucose transporter 1 and various glycolytic enzymes; HIF-1 also stimulates lactate dehydrogenase expression, promoting the conversion of pyruvate to lactate [24].

In addition to the glycolytic pathway, glutamine catabolism is a secondary source of lactate production in tumour cells. Upon entering the cell, glutamine is metabolised to α-ketoglutarate, which subsequently enters the mitochondria to participate in the citric acid cycle. When the citric acid cycle reaches oxaloacetate, it leaves the cycle in the form of malate and is converted into pyruvate by malate dehydrogenase, ultimately being converted into lactate [18].

Therefore, elevated lactate levels in tumour cells are not the result of a single metabolic event but rather the combined effect of enhanced glycolysis, mitochondrial metabolic reprogramming, activation of hypoxia signals, and oncogene-driven mechanisms [25]. On the one hand, signals from HIF-1α, MYC, KRAS and others can upregulate glucose transporters and various glycolytic enzymes [26], enhancing glucose uptake and glycolytic flux; on the other hand, increased LDHA expression promotes the conversion of pyruvate to lactate whilst simultaneously regenerating NAD^+^ to sustain continuous glycolysis. Concurrently, pyruvate dehydrogenase kinase (PDK) inhibits the entry of pyruvate into the tricarboxylic acid cycle, directing more carbon sources towards lactate production. In addition to glucose sources, glutamine breakdown, enhanced malate dehydrogenase activity, and restricted mitochondrial oxidative phosphorylation can further promote lactate accumulation. Consequently, lactate accumulation is not only a result of tumour metabolic reprogramming but also provides the material basis for subsequent lactylation modifications and DDR regulation.

### 2.2. Lactate Shuttling

Lactate shuttling is a key process in lactate signalling, involving a variety of physiological and pathological processes. Under normal conditions, glycolytic cells that actively release lactate may be referred to as “driver cells”, whilst cells that utilise lactate as a metabolic substrate and take up lactate may be referred to as “recipient cells”. A dynamic equilibrium is maintained between the two through the efflux and uptake of lactate, a process primarily mediated by MCTs [27]. There are several types of MCTs, each with a specific transport direction [17]. Cells with low intracellular lactate concentrations primarily utilise MCT1 to take up lactate, whereas cells with elevated intracellular lactate levels employ MCT4 to efflux excess lactate. MCT1 is widely distributed across various cell types, whereas MCT4 is primarily concentrated in cells with high glycolytic activity [28]. Intracellular lactate shuttling pathways include cytoplasm–mitochondria shuttling, peroxisomal shuttling, and other transport systems. Among these, cytoplasm–mitochondria lactate shuttling is considered to play a key role in the mitochondrial oxidation of lactate. The mitochondrial lactate oxidation complex (mLOC) model proposed by Brooks suggests that this process depends on MCT, its membrane-associated protein basigin (BSG or CD147), LDH, and cytochrome oxidase (COX) [29].

Lactate shuttling has emerged as a therapeutic target for various pathological processes, including cancer treatment; lactate dysregulation leads to abnormal lactate metabolism, which supports tumour initiation and progression. MCT1 and MCT4, along with their associated transmembrane glycoprotein CD147, are upregulated in tumour cells, causing an imbalance in the lactate shuttle system, which exerts oncogenic effects and promotes tumour drug resistance [30,31]. Currently, three modes of lactate shuttling are recognised in tumour cells: vascular endothelial cell shuttling, the reverse Warburg effect, and metabolic symbiosis.

**Figure 2:**
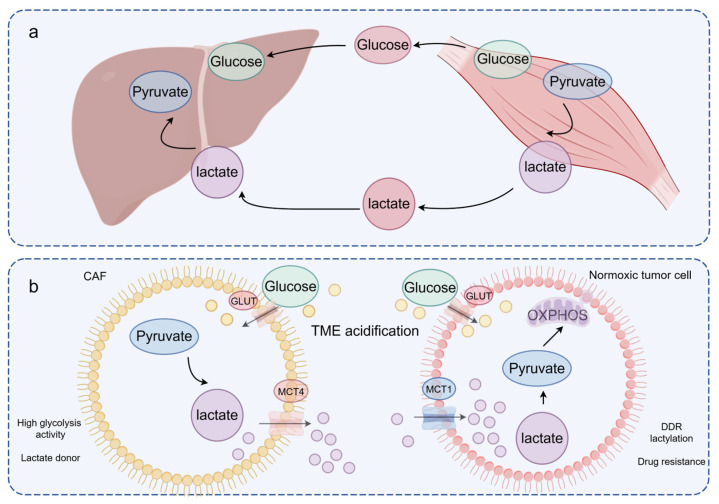
Schematic overview of lactate-mediated metabolic coupling. (**a**) In the glucose–lactate cycle, skeletal muscle converts glucose into pyruvate and lactate through glycolysis. Lactate enters the circulation and is transported to the liver, where it is reconverted into pyruvate and subsequently used for glucose production. The regenerated glucose is then released back into the bloodstream for use by peripheral tissues. (**b**) In the tumour microenvironment, glycolytic CAFs act as lactate donors by taking up glucose and producing lactate, which is exported via MCT4 and promotes extracellular acidification. Normoxic tumour cells import lactate through MCT1, convert it into pyruvate, and utilise it for OXPHOS. This metabolic symbiosis contributes to tumour adaptation, DDR-associated lactylation, and therapeutic resistance. Abbreviations: CAF, cancer-associated fibroblast; OXPHOS, oxidative phosphorylation; TME, tumour microenvironment; DDR, DNA damage response.

Vascular endothelial cell shuttling refers to the process whereby lactate produced by tumour cells is taken up by surrounding vascular endothelial cells, activating signalling pathways such as HIF-1α and NF-κB, and promoting the expression of pro-angiogenic factors such as VEGF. This process enhances the migration, proliferation and tubulogenesis capabilities of endothelial cells, driving tumour angiogenesis and providing support for tumour growth and metastasis. The reverse Warburg effect refers to tumour cells inducing neighbouring cancer-associated fibroblasts to undergo enhanced glycolysis, thereby producing and secreting large amounts of lactate. Subsequently, tumour cells take up this lactate via monocarboxylate transporters, convert it into pyruvate, and subject it to oxidative metabolism within the mitochondria, thereby obtaining energy and substrates for biosynthesis, which promotes tumour growth [32,33]. Metabolic symbiosis refers to the division of labour in lactate utilisation among cells in different regions within the tumour. Tumour cells in hypoxic regions primarily rely on glycolysis to produce lactate, which is then secreted via MCT4; conversely, tumour cells in better-oxygenated regions take up lactate via MCT1 and utilise it for oxidative phosphorylation to generate energy, thereby conserving glucose for hypoxic cells and maintaining the overall metabolic balance of the tumour [19] (Figure 2).

It is worth noting that MCT1/MCT4-mediated lactate transport not only influences the acidification of the tumour microenvironment but may also regulate lactate levels by altering intracellular lactate availability. Generally, MCT4 is highly expressed in tumour cells characterised by high glycolysis, hypoxia, or vigorous lactate production, and is responsible for effluxing excess lactate to prevent intracellular acidosis and sustain continuous glycolysis, whereas MCT1 is frequently involved in lactate uptake, enabling better-oxygenated tumour cells or stromal cells to utilise lactate as a substrate for oxidative metabolism. Consequently, differences in MCT1/MCT4 expression can determine intracellular lactate concentrations, lactyl-CoA production and protein lactylation levels in tumour cells, thereby potentially influencing the efficiency of DNA damage repair. Under the stress of chemotherapy, this lactate shuttle may further promote the formation of drug-resistant cell populations. On the one hand, hypoxic or hyperglycolytic tumour cells excrete lactate via MCT4, creating an acidic microenvironment that impairs drug uptake and the killing function of immune cells; on the other hand, MCT1-positive oxygenated tumour cells can uptake exogenous lactate, utilising it for oxidative phosphorylation to generate energy and providing ATP and metabolic intermediates for the DNA repair process following damage. Furthermore, increased lactate uptake may elevate intracellular lactylation levels, promoting enhanced function of DDR-related proteins or upregulation of DNA repair gene expression.

Consequently, lactate shuttling not only sustains metabolic symbiosis among tumour cells but may also promote the formation of cell subpopulations with enhanced DNA repair capacity and treatment tolerance by reshaping intracellular lactate availability and lactylation status.

### 2.3. The Process of Protein Lactylation and Its Regulatory Mechanisms

**Figure 3:**
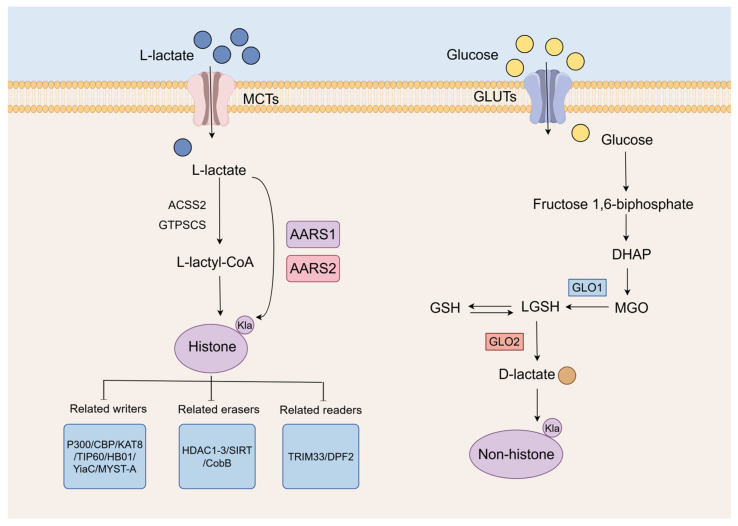
Mechanisms of lactylation regulation: histone lactylation and non-histone lactylation. The formation of lactylation primarily involves two pathways: L-lactate and D-lactate. Upon entering the cell, L-lactate is converted into L-lactyl-CoA by enzymes such as ACSS2 and GTPSCS, and subsequently mediates histone lactylation with the involvement of AARS1/2; the relevant “writing enzymes”, “erasing enzymes” and “reading proteins” jointly regulate this dynamic equilibrium. Conversely, methylglyoxal produced during glucose metabolism is converted into D-lactate via GLO1/GLO2 and the glutathione system, promoting non-histone lactylation, thereby reflecting the complexity of the lactylation sources and regulatory networks. Abbreviations: ACSS2, acyl-CoA synthetase short-chain family member 2; GTPSCS, GTP-specific succinyl-CoA synthetase; AARS1, alanyl-tRNA synthetase 1; AARS2, alanyl-tRNA synthetase 2, mitochondrial; Kla, lysine lactylation; P300/CBP, E1A binding protein p300/CREB-binding protein; TIP60, Tat-interactive protein 60 kDa; HBO1, histone acetyltransferase binding to ORC1; HDAC1–3, histone deacetylases 1–3; SIRT, sirtuins; TRIM33, tripartite motif-containing protein 33; DPF2, double PHD fingers 2; DHAP, dihydroxyacetone phosphate; MGO, methylglyoxal; GSH, glutathione; LGSH, lactoylglutathione; GLO1, glyoxalase 1; GLO2, glyoxalase 2.

The occurrence of lactylation depends on intracellular lactate levels and its conversion into a lactyl donor. The large amounts of lactate produced in tumour cells can influence epigenetic regulation within the cell nucleus in various ways. Firstly, as a small-molecule metabolite, lactate can form a concentration gradient within the cell and influence its availability within the nucleus through metabolic exchange between the cytoplasm and the nucleus. Since the nucleus is not a completely isolated metabolic compartment, lactate and its derivative metabolites that accumulate in the cytoplasm can influence enzymatic reactions within the nucleus, thereby participating in the regulation of chromatin modification. Consequently, the process by which lactate enters the nucleus or is converted into lactyl-CoA represents a key step in the translation of tumour metabolic states into signals regulating epigenetic and protein function. In the context of the DNA damage response, nuclear lactylation may not only alter chromatin accessibility and the transcription of DDR-related genes via histone lactylation but may also directly influence DNA damage recognition, repair complex assembly, repair pathway selection, and cellular sensitivity to chemotherapeutic agents by modifying non-histones such as NBS1 and XRCC1 [34,35]. The regulatory mechanisms of protein lactylation are shown in Figure 3.

#### 2.3.1. Mechanisms of Tumour Regulation via Histone Lactylation

Histones form nucleosomes with DNA, constituting the basic structural units of chromatin. Intracellular lactate can bind to lysine residues on histones, inducing lactylation. Lactate binds to histone lysine residues, causing their lactylation. Histone lactylation can epigenetically influence processes such as gene expression and DNA damage repair [36]. It plays a significant role in promoting the polarisation of macrophages towards an anti-inflammatory phenotype to participate in inflammatory responses, influencing the progression of neurodegenerative diseases, and facilitating immune evasion by tumour cells.

The KL-la pathway begins with the conversion of L-lactate to lactyl-coenzyme A under the catalysis of lactyl-coenzyme A synthases (ACSS2 and GTPSCS) [37,38]. Subsequently, lactyl-CoA is transferred to lysine residues under the action of lactate-modifying enzymes (such as lysyl acetyltransferases CBP/p300, KAT8, TIP60 and HBO1) [34,39,40]. Currently identified KL-la “erasers” include HDAC1–3, SIRT2 and SIRT3. Furthermore, lactate receptors and lactate transferases—namely alanine tRNA synthetases (AARS1 and AARS2)—have been identified within cells; these can directly bind lactate and catalyse global lysine lactylation.

Histone lactylation represents an important epigenetic mechanism by which cellular metabolic status is translated into chromatin-level regulation. Similarly to other lysine acylations, lactylation can neutralise the positive charge of lysine residues on histone tails, thereby weakening histone–DNA interactions and promoting a more relaxed chromatin conformation. This structural change may increase the accessibility of transcription factors, chromatin remodelling complexes and DNA repair proteins to specific genomic regions. In particular, lactylation at histone sites such as H3K18la has been associated with transcriptional activation, suggesting that histone lactylation functions not merely as a metabolic by-product but as a dynamic regulatory mark linking lactate accumulation to gene expression programmes.

In the context of DNA damage repair, histone lactylation may influence repair pathway choice by modulating chromatin accessibility and the recruitment of repair-associated factors. Open chromatin states generally facilitate damage recognition and repair protein loading, whereas compact chromatin can restrict access to DNA lesions. Therefore, lactate-driven histone lactylation may affect the balance between homologous recombination and non-homologous end joining by regulating the local chromatin environment surrounding DNA breaks. In tumour cells, where glycolysis-derived lactate is frequently elevated, enhanced histone lactylation may further reshape the transcriptional and epigenetic landscape, contributing to altered DNA repair capacity, therapy resistance and immune evasion. Thus, a detailed consideration of histone lactylation is essential for understanding how lactate metabolism participates in genome maintenance and disease progression.

#### 2.3.2. Tumour Regulatory Mechanisms of Non-Histone Lactylation

KL-la is an important lactylation modification occurring within cells. This modification is not limited to histones; various non-histones can also undergo lactylation [40]. It regulates protein function through covalent binding to lysine residues [41]. Non-histone lactylation involves several key proteins, including p53, PD-L1 and MRE11, and directly influences tumourigenesis, immune evasion and drug resistance [35,42,43].The interaction between ALDH1A3 and PKM2 promotes PKM2 tetramerisation, leading to lactate accumulation in glioma stem cells (GSCs) and subsequent lactylation at the XRCC1 K247 site [35]. Furthermore, in liver cancer stem cells (LCSCs), the mRNA and protein levels of glycolysis-related enzymes are significantly elevated, leading to enhanced glycolytic activity and increased intracellular lactate levels, thereby resulting in higher overall lactylation levels in LCSCs compared to hepatocellular carcinoma (HCC) cells [42]. Non-histone lactylation is closely involved in tumourigenesis and progression, particularly in mediating tumour resistance through DNA damage repair. Consequently, histone and non-histone lactylation may participate in the remodelling of the DDR at the levels of transcriptional regulation and protein function regulation, respectively.

### 2.4. Enzymatic Regulatory Mechanisms of Lactylation

#### 2.4.1. Potential “Writers”

Lactylating enzymes are primarily divided into two major categories: one comprises acyltransferases that utilise lactyl-CoA, whilst the other comprises AARS that utilise lactate and ATP. Lysyl acyltransferases (KATs) primarily function by participating in the acetylation process catalysed by acetyl-CoA; some KATs may also utilise lactyl-CoA to catalyse lactylation—a hypothesis that has been confirmed by in vitro experiments: p300 can catalyse lactylation using synthetically produced lactyl-CoA [36]. KAT5/TIP60, acting as an acyltransferase “writer”, mediates the lactylation of Vps34 at lysine 356 and lysine 781, thereby enhancing its binding to Beclin1, Atg14L and UVRAG [43]. The entire process is activated by the direct interaction between ULK1 and LDHA.

AARS encodes a class II acyl-tRNA synthetase, responsible for catalysing the linkage of L-alanine to tRNA during protein translation, an enzyme that catalyses lactylation using lactate and ATP as substrates [44,45]. Subsequently, the cytoplasmic subtype AARS1 was also found to possess lactyltransferase activity. Given the importance of “writing enzymes”, subsequent studies should delve deeper into the upstream regulatory mechanisms of lactyl-CoA and lactate-dependent lactylation, as well as the substrate specificity of different lactyltransferases.

#### 2.4.2. Potential “Erasers”

Another key component of lactylation modification is the “eraser”; the first to be identified were histone deacetylases (HDACs, including HDAC1–3) and sirtuins (SIRTs, including SIRT1–3) [46,47], which primarily act on L-lactylation [12,36]. HDAC1–3 can also exert de-lactylation activity on D-lactylation.

Moreno-Yruela et al. conducted an in vitro screening of all 18 lysine deacetylases (KDACs), and the results showed that HDAC1, HDAC2 and HDAC3 possess de-lactylase activity on both peptides and histones, whereas SIRT1, SIRT2 and SIRT3 exhibit this activity only on histones [46]. In cell-based studies, HDAC inhibitors and class I HDAC inhibitors have been found to increase overall histone lactylation levels [46]; however, SIRT inhibitors and class IIa HDAC inhibitors did not exhibit such effects [36].

#### 2.4.3. Current Status of “Reader” Research

Readers are effector proteins capable of specifically recognising and binding to histones in a PTM-specific manner. Recognition proteins play a crucial role in regulating DNA processes (such as transcription, replication, recombination and repair). The bromo domain is a class of protein-interaction domains initially identified as lysine acetylation recognition modules. Given the chemical structural similarity between lactylation and acetylation, it has been hypothesised that bromo domains may also recognise lactylation modifications. Smith et al. conducted a systematic screening of 28 bromo domains to identify potential lactylation-recognising proteins; the results revealed that only the PHD-bromo domain of TRIM33 was capable of binding to histone peptides bearing lactylation modifications. Apart from bromo-domains, it remains unclear whether other families of histone acetylation-recognising proteins are capable of recognising lactylation modifications.

## 3. Lactylation-Mediated Remodelling of DNA Damage Repair

**Figure 4:**
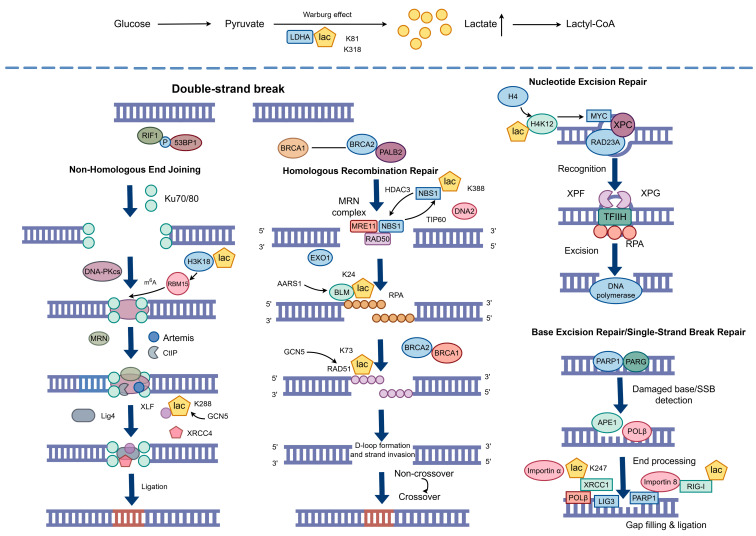
Lactylation and DNA damage signalling pathways: DSB repair (NHEJ), non-DSB repair (NER, BER/SSBR). Lactylation promotes the maintenance of genomic stability through multiple DNA damage repair pathways. As shown in the figure, during homologous recombination, lactylation of NBS1, BLM, RAD51 and related histone sites enhances DNA end processing, strand invasion and the repair transcription programme; in non-homologous end joining, XLF lactylation promotes its synergy with Ku80; in nucleotide excision repair, H4K12 lactylation upregulates RAD23A via MYC; in BER/SSBR, XRCC1 lactylation promotes nucleotransposition and enhances repair complex assembly. Abbreviations: HR, homologous recombination; NHEJ, non-homologous end joining; NER, nucleotide excision repair; BER, base excision repair; SSBR, single-strand break repair; DSB, double-strand break; MRE11, meiotic recombination 11 homologue 1; RAD50, RAD50 double-strand break repair protein; NBS1, Nijmegen breakage syndrome 1; HDAC3, histone deacetylase 3; GCN5, general control non-derepressible 5; BLM, Bloom syndrome protein; DNA2, DNA replication helicase/nuclease 2; RPA, replication protein A; RAD51, RAD51 recombinase; BRCA2, breast cancer type 2 susceptibility protein; Ku80, X-ray repair cross-complementing protein 5; XLF, XRCC4-like factor; RAD1, RAD1 checkpoint DNA exonuclease.

Metabolic reprogramming and genomic instability are fundamental characteristics of cancer. A growing body of evidence suggests that these two processes are functionally closely linked. Lactylation also plays a key role in DNA repair, a core process that maintains genomic stability. Lactylation is increasingly recognised as a key regulator of histone and non-histone modifications, thereby contributing to the development of treatment resistance [48,49]. Enhanced glycolysis in tumour cells has been linked to the regulation of key DNA repair pathways mediated by lactylation, with DNA repair processes linking tumour resistance to the lactylation modification process [50,51]. The regulatory mechanism linking lactylation to DNA damage and drug resistance is shown in Figure 4. We have summarised the links between lactylation sites and the types of DNA repair and tumour resistance in Table 1.

In addition, the role of DNA glycosylases in determining repair pathway preference should be considered, particularly in BER [61]. DNA glycosylases initiate BER by recognising and removing damaged bases, thereby generating distinct repair intermediates that influence subsequent processing steps. Depending on lesion complexity, strand context, and the coordinated action of downstream factors such as APE1, DNA polymerase beta, FEN1 and PCNA, BER may proceed via short-patch BER (SP-BER) or long-patch BER (LP-BER) [62,63,64,65]. Therefore, DNA glycosylases are not only lesion-sensing enzymes but also important contributors to LP/SP pathway choice.

### 3.1. BER/SSBR and Lactylation

SSBs are primarily resolved by the single-strand break repair (SSBR) pathway, which is closely coordinated with BER and shares several key repair factors with BER. By contrast, NER and mismatch repair (MMR) mainly address distinct types of DNA lesions, although their repair processes may generate transient single-strand incision intermediates.

Base excision repair results in the replacement of only 1 to 10 nucleotides (no more than 10) by short or long patches. The choice between short-patch and long-patch base excision repair is influenced by cellular state and context, the type of damage, and the levels of endogenous and exogenous substances. The importance of base excision repair lies not only in ensuring genomic stability; its dysregulation also increases the risk of cancer, age-related diseases and other serious conditions. However, base excision repair is not an isolated repair pathway but rather a vital component of the larger DNA damage repair machinery; it forms a network in synergy with other pathways and may be regulated by them via feedback mechanisms. Furthermore, once XRCC1 is recruited to DNA break sites by PARP1 (poly ADP-ribose polymerase 1), it undergoes various PTMs, such as phosphorylation, ubiquitination and Kla [66,67]. These modifications regulate its core functions in SSB and DSB repair. Given that genomic instability is a hallmark of cancer cells, they counteract the accumulation of DNA damage by enhancing DNA repair pathways [35]. However, the excessive activation of these repair mechanisms can boost repair capacity, ultimately leading to cancer cell resistance to treatment.

RIG-I (DDX58) undergoes lactylation within the nucleus, a process mediated by the acetyltransferase PCAF. This enhances the nuclear translocation of lactylated RIG-I in an importin 8-dependent manner. Nuclear-localised RIG-I interacts with PARP1 and attenuates its activity, thereby inhibiting DNA damage repair [55]. The interaction between ALDH1A3 and PKM2 enhances PKM2 tetramerisation and promotes lactate accumulation in GSCs. Studies have shown that XRCC1 undergoes lactylation at lysine 247 (K247). Lactylated XRCC1 binds more strongly to importin α, thereby promoting increased nuclear transport of XRCC1 and enhancing DNA repair capacity [35].

In addition to nuclear BER, mitochondrial BER may also be affected by lactylation-dependent regulation. Mitochondrial DNA is highly susceptible to oxidative damage due to its proximity to the electron transport chain, and BER represents the major pathway responsible for repairing mtDNA lesions. Several enzymes involved in BER, such as DNA glycosylases, APE1, DNA polymerase γ, FEN1 and DNA ligase III, coordinate lesion recognition, end processing, gap filling and ligation within mitochondria. Although direct evidence linking lactylation to mitochondrial BER remains limited, lactate accumulation may reshape mitochondrial repair capacity through metabolic and post-translational mechanisms [68,69]. For example, altered lactate metabolism can influence mitochondrial redox homeostasis, ROS generation and NAD^+^/NADH balance, thereby affecting mtDNA damage burden and the activity of BER enzymes. Potential lactylation of mitochondrial or mitochondria-associated repair proteins may further regulate their stability, localisation or enzymatic activity, suggesting a possible connection between lactylation and mtDNA repair efficiency.

### 3.2. NER and Lactylation

Nucleotide excision repair is a complex process, as it must address complex lesions, including bulky adducts and cross-links; this pathway comprises two sub-pathways: transcription-coupled nucleotide excision repair (TCR-NER) and genome-wide nucleotide excision repair (GGR-NER). Such DNA damage is often caused by alkylating agents or cross-linking agents. Defects in nucleotide excision repair are frequently associated with various diseases. Research over the past few decades has elucidated this repair process: in TCR-NER, an RNA polymerase stalled by damage constitutes the initial step in recognising DNA damage, subsequently recruiting the CSB/ERCC6 complex, which in turn recruits the CSA/ERCC8 complex.

The target gene RAD23A is significantly upregulated in cells resistant to niraparib; inhibition of RAD23A restores drug sensitivity. Lactate accumulation induces lactylation of the lysine residue H4K12; H4K12la activates the niraparib super-enhancer (SE) and RAD23A expression via the MYC transcription factor, thereby enhancing DNA damage repair capacity and promoting resistance in ovarian cancer cells [56].

### 3.3. NHEJ and Lactylation

Two main DSB repair pathways have been reported to date: HR and NHEJ. The NHEJ pathway is capable of repairing DSBs throughout the cell cycle. In the classical NHEJ pathway, a heterodimer formed by Ku70 and Ku80 first binds to the broken DNA ends, subsequently recruiting DNA-PKcs (DNA-dependent protein kinase catalytic subunits). DNA-PKcs, members of the phosphoinositide 3-kinase (PIKK) kinase family, bring the two broken DNA ends closer together and recruit end-processing-related enzymes such as Artemis, PNKP (polynucleotide kinase/phosphatase), APE1 (AP endonuclease 1) and Tdp1 (tyrosyl-DNA phosphatase 1), among other end-processing-related enzymes, which in turn recruit the XRCC4-XLF-LIG4 complex [70,71].

Hypoxia increases lactate production via LDHA, which in turn induces H3K18la modification catalysed by KAT2B, thereby activating RBM15 transcription; RBM15 utilises its SPOC domain to perform m6A modification on IGFBP3 mRNA, enhancing its stability and increasing IGFBP3 protein levels; IGFBP3 forms a complex with phosphorylated EGFR and phosphorylated DNA-PKcs and is transported into the nucleus, enhancing DNA repair capacity and mitigating cisplatin-induced DNA damage [57].

Lactate is transported from TAMs to GSCs via MCT4–MCT1. TAMs supply lactate to GSCs, promoting GSC proliferation and inducing lactylation at lysine 317 (K317) of the non-homologous end-joining protein KU70, thereby inhibiting the cGAS–STING signalling pathway and reshaping the immunosuppressive TME [58]. Lactate derived from glycolysis promotes the lactylation of XLF at the K288 site within its Ku-binding motif (X-KBM), thereby regulating NHEJ. Mechanistically, DNA damage triggers ATM-mediated phosphorylation of GCN5, enhancing the interaction between GCN5 and XLF, as well as XLF lactylation, thereby strengthening the binding of XLF to Ku80, the recruitment of XLF to DSB sites, and NHEJ efficiency [60]. Screening identified Lys81 and Lys318 of LDHA as its key lactylation sites, and AARS1 as the lactyltransferase mediating this lactylation. Mass spectrometry analysis revealed that numerous proteins involved in NHEJ, including FEN1, XRCC5 and XRCC6, may be regulated by lactylation [59].

### 3.4. HR and Lactylation

Compared with the NHEJ pathway, the HR pathway is more conservative and error-free due to its reliance on the presence of sister chromatids. However, this characteristic also restricts the HR pathway to DSB repair only during the S and G2 phases of the cell cycle when sister chromatids are present. In the HR pathway, multiple proteins must act in concert. Following DSB formation, under the action of specific nucleases (Mre11-Rad50-Nbs1, i.e., the MRN complex), NBS1 binds and the MRN complex directs ATM to the DSB site; ATM is subsequently activated and, through ATM-dependent phosphorylation of H2AX (γH2AX), further transmits the DNA damage signal, triggering HR-dependent repair [72,73]. The 5′ end of the DSB is excised, forming a 3′ single-stranded DNA (ssDNA) end. Subsequently, the ssDNA is wrapped by RPA and replaced by Rad51, forming a Rad51 nucleofilament structure. Intermediary proteins such as RAD52 and BRCA2 participate in this process [74,75]. Subsequently, with the assistance of PALB2 and rad51ap1, the Rad51 nucleofilament binds to homologous double-stranded DNA, forming a D-loop structure [76,77].

Lactate-induced lactylation promotes resistance to anthracycline drugs by regulating HR repair. Under chemotherapeutic stress, the helicase BLM undergoes extensive lactylation at lysine 24 (Lys24), mediated by AARS1. Mechanistically, the high lactylation of BLM enhances its protein stability by inhibiting MIB1-mediated ubiquitination and strengthening its interaction with DNA repair factors, thereby promoting DNA end resection and HR repair [52].

Malate dehydrogenase 2 (ME2) metabolises malate derived from glutamine into pyruvate, thereby promoting lactate production and chemoresistance in ovarian cancer. The mechanism is as follows: chemotherapy treatment reduces the expression of glucose transporters in cancer cells, impairing glucose uptake; the resulting decrease in intracellular glucose levels triggers ACAT1-mediated acetylation of lysine 156 (K156) in ME2, thereby enhancing ME2 enzymatic activity and promoting lactate production from glutamine; ME2-derived lactate primarily acts by promoting the lactylation of proteins involved in homologous recombination repair [54].

Lactylation directly regulates the composition of the meiosis-recombination 11 MRE11–RAD50–NBS1 (MRN complex), which is a key participant in the HR process. For example, TIP60-mediated lactylation of NBS1 at K388 is crucial for MRN complex assembly. TIP60 acts as the NBS1 lysine lactylation “writer,” i.e., the enzyme responsible for the lactylation of NBS1 at the K388 site, whilst HDAC3 acts as the deacetylase for NBS1 [34]. Ovarian cancer is a highly lethal gynaecological malignancy that frequently develops platinum resistance. Studies have found elevated levels of histone lactylation in platinum-resistant ovarian cancer, particularly H3K9la. Mechanistically, H3K9la directly activates the expression of RAD51 and BRCA2, promoting HR repair; furthermore, RAD51K73la enhances HR repair, thereby conferring cisplatin resistance. H3K9la and RAD51K73la share the same upstream regulator, GCN5 (which utilises lactyl-coenzyme A as a substrate) [53].

## 4. Lactylation Holds Significant Therapeutic Potential

### 4.1. MCT Inhibitors

**Figure 5:**
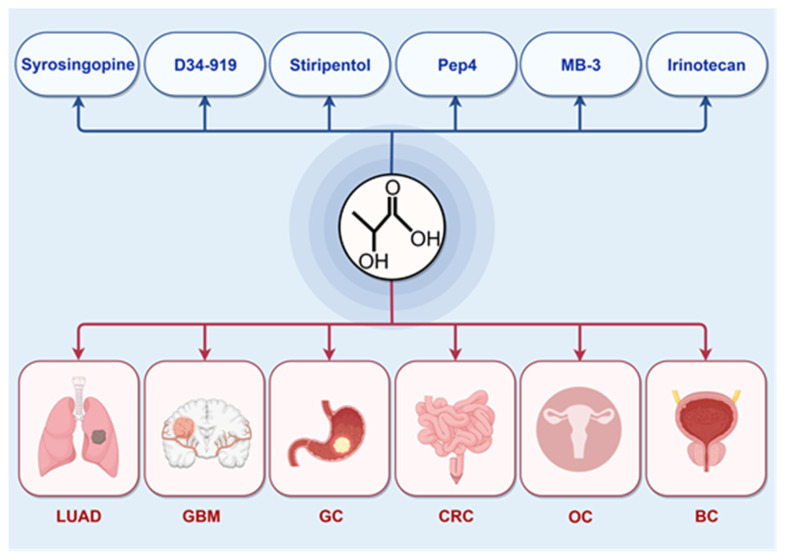
Lactylation links DNA damage to drug resistance in various tumours and represents a potential target for targeted drugs. Targeting lactate metabolism and lactylation regulation offers new strategies for cancer treatment. The figure illustrates a range of therapeutic agents and their potential indications, including the MCT inhibitors syrosingopine and D34-919, the LDH-related inhibitor stiripentol, and agents targeting the lactylation pathway such as Pep4, MB-3 and irinotecan. These agents may enhance treatment sensitivity in lung adenocarcinoma, glioblastoma, gastric cancer, colorectal cancer, ovarian cancer and bladder cancer by reducing lactate accumulation, inhibiting lactylation or impairing DNA repair capacity.

Targeting MCTs has a significant impact on metabolic co-production, with MCT1 and MCT4 being potential targets for the treatment of solid tumours [78,79]. There is a sound rationale for combining the MCT1/4 inhibitor syrosingopine with PARPi in the treatment of lung adenocarcinoma. Inhibiting DNA damage repair by suppressing PARP1 activity enhances the sensitivity of LUAD cells to PARP inhibitors (PARPi) and significantly potentiates the therapeutic effect of olaparib in a mouse LUAD model [55]. The MCT1 inhibitor AZD3965 has completed a Phase I clinical trial for advanced solid tumours, diffuse large B-cell lymphoma and Burkitt’s lymphoma. The effects of lactylation inhibitors on various tumours are shown in Figure 5.

### 4.2. LDHA Inhibitors

The LDHA inhibitor stiripentol reduces lactate levels, inhibits NBS1 K388 lactylation, impairs DNA repair efficiency, and reverses chemotherapy resistance [34]. Combination therapy with the LDHA inhibitor (stiripentol) and the EGFR inhibitor (gefitinib) synergistically reverses cisplatin resistance both in vitro and in vivo [57]. To date, a variety of small molecules have been identified that exhibit significant LDHA inhibitory activity and anticancer activity; consequently, alternative anticancer therapies have been developed that inhibit cancer cells by selectively suppressing tumour glycolysis [80].

### 4.3. ACAT1 Inhibitors

ACAT1 inhibitors include K-604 and Avasimibe. Targeting ACAT1 to inhibit ME2 acetylation effectively reduces chemotherapy resistance in both in vitro and in vivo ovarian cancer models [54]. Targeting the ACAT1-ME2 axis offers a promising strategy for overcoming platinum-based chemotherapy resistance.

ACAT1 inhibitors are potent agents in cancer immunotherapy. ACAT1 inhibitors have been shown to synergistically enhance the antitumour effects of anti-PD-1 antibody therapy or CAR-T cell therapy in mouse tumour models [81].

### 4.4. Irinotecan

Irinotecan demonstrates synergistic effects and safety by targeting BLM lactylation and inhibiting HR repair, effectively mitigating resistance to anthracyclines. The irinotecan liposome combined with the EPI regimen represents a feasible and safe treatment strategy for patients with recurrent anthracycline-resistant bladder cancer [52].

This study considers the clinical combination of this drug with other anticancer agents, such as novel drug formulations (e.g., lipid-based formulations, dendrimers and nanoparticles). It also elucidates the key mechanisms underlying tumour cell resistance to the active metabolite ethyl-10-hydroxy-s-n-38 (SN-38) [82].

### 4.5. GCN5 Inhibitors

GCN5 inhibitors significantly enhanced the antitumour efficacy of cisplatin in ovarian cancer PDX models. Studies have confirmed the key role of histone and RAD51 lactylation in HR repair and platinum resistance and have proposed a potential therapeutic strategy that may overcome platinum resistance and improve the prognosis of ovarian cancer patients [53].

DC_G16 is a novel GCN5 inhibitor featuring a 1,8-acridinedione scaffold, and it holds considerable therapeutic potential in clinical settings [83]. Its distinctive chemical structure also provides a useful basis for further optimisation to improve potency, selectivity, and pharmacokinetic performance. Given the central role of GCN5 in chromatin remodelling and cancer-associated gene regulation, DC_G16 may serve as a promising lead compound for the development of targeted therapies with potential clinical applications [83].

### 4.6. D34-919

D34-919 is a drug that inhibits the activation of pyruvate kinase by ALDH1A3; it is capable of reversing chemoradiotherapy resistance in patients with ALDH1A3-overexpressing GSCs. D34-919 specifically antagonises the protein–protein interaction between ALDH1A3 and PKM2. Validated through in vitro and in vivo experiments, as well as in GSC organoids, this small-molecule inhibitor demonstrates good therapeutic efficacy with few side effects [35].

### 4.7. Lactylation Inhibitory Peptide: Pep4

A peptide inhibitor specifically targeting XLF K288 lactylation, when combined with 5-fluorouracil, exhibits synergistic killing of colorectal cancer cells in PDX models with high XLF lactylation levels [60]. This study reveals that the GCN5–XLF lactylation axis is a key regulator of NHEJ, and targeting XLF lactylation is expected to enhance the efficacy of chemotherapy.

Pep4 exhibits a marked inhibitory effect on the lactylation of XLF at K288; Pep4 itself is also subject to lactylation. When lactylated, Pep4 binds to Ku80, thereby competitively inhibiting the lactylation of endogenous XLF and its interaction with Ku80, ultimately impairing XLF function. Treatment with Pep4 enhances the sensitivity of cancer cells to 5-FU therapy [60].

### 4.8. MB-3

Research on the GCN5 inhibitor MB-3 (α-methylene-γ-butyrolactone) has revealed that it enhances the response of ovarian cancer to CDDP by inhibiting the enrichment of H3K9la and RAD51K73la. MB-3 inhibits the enrichment of H3K9la at the RAD51 and BRCA2 promoter regions, thereby downregulating their mRNA expression. Combination therapy with MB-3 in patient-derived xenograft (PDX) models has been shown to increase sensitivity to CDDP in specific ovarian cancer cases and even reverse drug resistance [53].

## 5. Prospects for Future Research

It is worth noting that there is a strong link between lactylation-mediated DNA repair remodelling and resistance to cancer treatment. Therapies such as radiotherapy, platinum-based drugs [84,85], PARP inhibitors and topoisomerase inhibitors all rely on the accumulation of DNA damage to induce tumour cell death, whereas tumour cells can counteract this therapeutic pressure by enhancing their DNA repair capacity [35,86]. Lactate accumulation and lactate-induced lactylation are integral components of this adaptive process. For example, lactylation of key repair proteins such as NBS1, BLM, RAD51, XRCC1 and XLF can enhance the corresponding DNA repair pathways, thereby reducing the sensitivity of tumour cells to DNA damage therapy. Consequently, targeting lactylation may represent a new strategy for overcoming treatment resistance. Compared to simply inhibiting DNA repair proteins, interfering with lactate production, transport, or the “writing/erasing” enzymes involved in lactylation may weaken the repair adaptability of tumour cells at the metabolic source.

From a translational therapy perspective, lactylation-related targets hold significant development potential [87,88]. Firstly, targeting the lactate metabolic pathway is the most direct strategy [13,89]. LDHA inhibitors can reduce lactate levels, thereby inhibiting NBS1 K388 lactylation, impairing DNA repair efficiency and reversing treatment resistance. MCT1/4 inhibitors can block transmembrane lactate transport, not only affecting lactate supply within tumour cells but also disrupting the metabolic symbiosis between tumour cells and immune or stromal cells. AARS1, GCN5/PCAF, TIP60 and the SIRT family may be involved in the lactylation or de-lactylation of different substrates, respectively. Inhibiting AARS1-mediated BLM lactylation, GCN5-mediated RAD51 or XLF lactylation, and modulating the TIP60-HDAC3-regulated balance of NBS1 lactylation may all enhance the therapeutic efficacy against DNA damage. Furthermore, site-specific lactylation-inhibiting peptides or small-molecule interventions warrant attention. For instance, an inhibitory peptide targeting XLF K288 lactylation, in combination with chemotherapy, has demonstrated synergistic anti-tumour potential, suggesting that site-specific interventions may offer greater selectivity and lower systemic toxicity. The influence of CEL (Nε-carboxyethyllysine)-related modifications on the selection of DNA damage repair pathways should be discussed within the context of lactate metabolic reprogramming and the regulation of protein lactylation. Although CEL and lysine lactylation differ in their chemical origins and modification mechanisms, both reflect changes in metabolic state and may influence chromatin structure, the recruitment of repair factors, and the HR/NHEJ balance. Consequently, elucidating the relationship between CEL-related modifications and lactylation will contribute to a more comprehensive understanding of the molecular mechanisms by which metabolism regulates the selection of DNA damage repair pathways [90].

However, lactylation-targeted therapies still face multifaceted challenges. Firstly, lactylation is extensively involved in the physiological regulation of normal cells, including immune responses, tissue repair, stem cell function and metabolic homeostasis. Consequently, systemic inhibition of lactate production or lactylation modifications may lead to off-target effects and safety concerns. Distinguishing between tumour-cell-dependent lactylation and physiological lactylation in normal tissues is a key issue that must be addressed in future drug development. Secondly, tumour cells exhibit high metabolic plasticity. When LDHA, MCTs or the glycolytic pathway are inhibited, tumour cells may maintain lactate supply and redox balance through glutamine metabolism, fatty acid oxidation, mitochondrial oxidative phosphorylation or other compensatory pathways. For example, ME2-mediated lactate production from glutamine can underpin chemotherapy resistance. Consequently, inhibition of a single metabolic target may prove difficult to sustain long-term; multi-pathway combined interventions or dynamic adaptive therapies may hold greater clinical value. Thirdly, many current studies on lactylation remain at the cellular and animal model stage, lacking validation with large-scale clinical samples and support from prospective clinical trials. Future research is needed to further clarify whether lactylation levels, site-specific modifications, and the expression of related enzymes can serve as biomarkers for predicting treatment response and the risk of resistance.

In the context of immunotherapy, lactate-mediated remodelling of DNA repair also warrants further investigation. On the one hand, DNA damage repair capacity influences tumour mutational burden, chromatin fragment release and cGAS-STING pathway activity, thereby modulating anti-tumour immune responses. On the other hand, lactate accumulation can suppress effector T-cell function, promote Treg cell maintenance and induce immunosuppressive polarisation of macrophages. The finding that KU70 lactylation inhibits cGAS-STING signalling suggests that lactylation may promote immune evasion via both DNA damage repair and immune signalling pathways. Therefore, combining immune checkpoint inhibitors with lactate metabolism inhibitors, MCT inhibitors, or key lactylase inhibitors may help break through immune suppression barriers and improve immunotherapy response rates. Future research should further clarify which tumour types, lactate modification profiles, and immune microenvironment states are most suitable for such combination therapies.

Overall, lactate modification provides a new theoretical framework for understanding the link between tumour metabolic reprogramming and DNA damage repair. Lactate accumulation not only remodels the expression of repair-related genes via histone lactate modification but also directly regulates the stability, localisation, interactions, and function of DNA repair proteins through non-histone lactate modification. Lactylation-mediated remodelling of BER/SSBR, NER, HR and NHEJ collectively contributes to the maintenance of genomic stability in tumour cells, as well as to treatment resistance and immune evasion. In the future, with the continuous development of lactylation detection technologies, site-specific intervention tools and clinical translational research, targeting lactylation is expected to become a key direction for overcoming treatment resistance to DNA damage, optimising combination therapy regimens and achieving personalised anti-tumour treatment.

## Figures and Tables

**Table 1:** Lactate-induced DNA damage sites in the development of drug resistance in tumours.

Disease	Lactylation-Modified Genes and Sites	Writer/Eraser	Clinical Significance or Potential Treatment	DNA Repair Signalling Pathways
Bladder cancer	*BLM* (Lys24)	AARS1	Liposomal irinotecan combined with EPI regimen for the treatment of recurrent anthracycline-resistant bladder cancer	HR repair [52]
Ovarian cancer	RAD51 (K73la)	GCN5	RAD51K73la enhances HR repair, thereby conferring cisplatin resistance; GCN5 inhibitors significantly enhance the antitumor effect of cisplatin in ovarian cancer PDX models.	HR repair [53]
Gastric cancer	*NBS1* (K388)	TIP60/HDAC3	The LDHA inhibitor stiripentol reduces lactate levels, inhibits NBS1K388 lactylation, impairs DNA repair efficiency, and reverses chemotherapy resistance.	HR repair [34]
Ovarian cancer	/	/	The critical role of ME2-mediated acetylation in glutamine-derived lactate production in chemotherapy resistance.	HR repair [54]
Lung adenocarcinoma	*RIG-I*	/	The combination of the MCT1/4 inhibitor syrosingopine with PARP inhibitors for the treatment of lung adenocarcinoma provides a rational basis.	BER/SSBR [55]
Ovarian cancer	H4K12la	/	H4K12la activates the niraparib super-enhancer (SE) and *RAD23A* expression via the MYC transcription factor, thereby enhancing DNA damage repair capacity and promoting drug resistance in ovarian cancer cells.	NER [56]
Glioblastoma	*XRCC1* (K247)	/	D34-919 combined with chemoradiotherapy significantly enhances apoptosis in GBM cells.	BER/SSBR [35]
Bladder cancer	H3K18la	KAT2B	Combination therapy with an *LDHA* inhibitor (stiripentol) targeting this axis and an EGFR inhibitor (gefitinib) synergistically reverses cisplatin resistance in vitro and in vivo.	NHEJ repair [57]
Glioblastoma	KU70 (K317)	/	Inhibition of lactate transport or targeting of KU70 lactylation, in combination with immune checkpoint blockade, demonstrates synergistic therapeutic benefits.	NHEJ repair [58]
Lung adenocarcinoma	*LDHA* Lys81 and Lys318	AARS1	Numerous proteins involved in non-homologous end joining (NHEJ), including *FEN1, XRCC5, and XRCC6*, may be regulated by lactylation.	NHEJ repair [59]
Colorectal cancer	*XLF* within its Ku-binding motif (X-KBM) K288la	GCN5	A peptide inhibitor of XLF K288 lactylation, in combination with 5-fluorouracil, synergistically kills colorectal cancer cells in PDX models with high XLF lactylation.	NHEJ repair [60]

## Data Availability

No new data were created or analysed in this study.
